# Dental Education

**Published:** 1866-06

**Authors:** W. H. Gates

**Affiliations:** Louisville


### DENTAL EDUCATION.

BY W. H. GATES.

Read before the Central States Dental Association.

Gentlemen:—The dentist, like other men, is in search of happiness. His vocation affords him either real or fancied pleasure. It can be real only as it is reciprocal. Dispensing good, it is reflected back into his own heart and life. Governed by impulses or opinions, true or deceptive, he pursues that path by which he hopes to realize success.

As water, on the dividing ridge, flows one way to the Atlantic—the other to the Pacific, so he may approach the east for light, or, guided by intuition, make his own path as a pioneer through the primitive shadows of the forest.

Along one route he approaches with diffidence, for the light reveals to him his defects, and he struggles with difficulties that are truly formidable; but inspired by the love of truth, now flashing round him, his energy arises with his ardor and he scales them with a perseverance, skill and power that surprises himself and excites the admiration of others. As he advances, time brings to him its true rewards—fewer thorns, more flowers appear — fortune extends her favors and fame, aye, good substantial fame will bid him welcome.

On the other side you see the man who trusts, as he says, in his "natural genius." As his face is turned from the light he naturally sees his own shadow, and generally, somewhat magnified. If he sees aught else, it is a vision of gold —some California which he doubts not soon to reach, by *a short route.*

For a time he is flattered by success. But his assurance is like the complacency of the invader; he has tread upon sacred ground and trifled with the misfortunes of his fellows. They recoil upon him; he can only proceed by strategy. He must appeal to prejudice, and rely upon ignorance to shield him, or fly to another field. In this manner, by a succession of movements, or devices, he *may* realize his golden vision, for even "the wicked shall prosper." But what then is his fame, and where his real happiness? He has made speculation of misfortune, and trifled with inestimable jewels—the gifts and handiwork of God. Can such reckless sacrilege bring peace of mind? Sooner will the thief his gains enjoy. He simply takes the purse, but this arch villain under the guise of good, makes robbery of jewels also, which the highest art or the most boundless wealth can not truly replace.

This association has honored me by placing in my hands a theme of such great weight as Dental education. But it was simply a mistake, for it is a mountain I can not measure—a sea, the pearls of whose depths I can not bring to light; but if, by a few surface thoughts, I can inspire one wavering brother to search in its bright waters, I am content.

Education is unquestionably the basis of all true excellence in the practical as well as theoretical employments of life; for it places at the door of each the compiled, invaluable experience of the past and present.

All truly worthy men, it may be said, however great or various their talent or skill, are modest and deferential. They respect the opinions and experience of others. Such men have met here to-day, and whether that experience be bound up in book form, as the legacies of those who, though dead, yet live in them, or whether it fall upon his ear to-day from living lips, they deem it a great privilege. And such it is, for here, avoiding the tedious and consuming labor of personal experience, we can start from that higher point to which they have attained, and rise higher and still higher in the scale.

Thus education, or science, which is simply a systematic collection of facts, is of the very first importaance to Art which is confined strictly to exemplifying or appropriating those facts in practice. If then, education comprises the benefits of experience and those already collected facts the knowledge of which constitutes our reputation and directs our skill who does not covet it? Who would not make his mind a storehouse of science that it might be ready at any time when called for? How humiliating, after permitting ourselves to be called "doctor" which signifies "learned" to be obliged to acknowledge or even feel that we are *not* learned—not able, it may be, to explain to our patient some simple principle which, as a cause, has produced injurious effects.

Let us then consider some of the benefits that accrue to our art from education. Education is knowledge, and its pursuit, by the exercise of our senses. If the eye brings us knowledge by what we see, so the ear by what we hear. The five senses are but as so many individuals thirsting for knowledge, and each, to a great extent, independent of the others.

That consideration which I esteem of the first importance in our art is skill. Talent is a gift, but skill is the child of education. It is the inseparable product of two of our senses. Tho eye measures, classifies, adjusts, directs; the touch executes. Now in proportion to the science in the eye, and the art in the hand, will be the beauty and perfection of the skill which they produce. But if the eye, which communicates the science from the brain—the storehouse of fact—has no storehouse there what of the art? It is like an eagle without a head—an army without a general—floundering in blood and chaos.

Science and art must go hand in hand to attain true success in our profession. Science is like the engine standing with its train of precious freight, ready to fly away to its destination; but art is the power and motion that will carry it there. If the science is good there will be no explosion—no uncoupling—no running off the track—nothing lost overboard. All is safe and happy, and, from his destination, the passenger looks back with rejoicing and gratitude for the benefit he has received.

Among the other benefits which science, as education is commonly understood to signify, confers, is consideration. Sensible people necessarily set a high estimate upon those organs that so greatly involve their comfort, health, personal appearance and pleasure. They will not, knowingly, permit them to be trifled with; and he who will truly qualify himself, by all the means attainable, to preserve their beauty and usefulness, will learn and other things being equal, will obtain, not alone a *monied* compensation but that delightful gratitude which a true service rendered always inspires.

Your reputation, too, will be like a genuine bank note with science and art modestly, but most beautifully interwoven on its face.

The people will take it up and circulate it until you can hardly supply the demand for that *real* coinage of your skill which they esteem more valuable than gold; for they feel that you are giving them not only the benefit of your own and the experience of others in the past, but in the present, by constant communication with the world's advancing thoughts and discoveries.

Thus as an exponent of good deeds and purposes, and crowned with honors and, thus far, with that true happiness for which we seek, the educated dentist is triumphant on the road to fortune and true fame.

But without this science your note is counterfeit. It is not beautiful; it is not attractive. And, though it sometimes passes in strange places, for want of a close examination, yet it will be treated with suspicion and finally be returned with imprecations on your hands. Shall I picture the end of such a man? He deserves not our notice, let us draw the veil over his obscurity.

But, to be brief, you say, how shall we obtain this education? Oh! how is any great or good end secured but by

*earnest effort*. Every true man has in his breast, a sublime courage that rejoices in the conflict with difficulties. But you are not left single-handed in the struggle. *That* is your trouble now. You are attempting to accomplish *without* assistance that which is difficult indeed, by *united* effort. Come, then, and unite with use. We will encourage, enlighten and sustain each other. Look also upon the shelves of science. There are our master-spirits with the free offering of the accumulated treasures of their life-long labors.

Look again, in your own Great West, where a Cincinnatus was remembered, or, on the Eastern Slope where a Franklin was buried, or, where, in the foundations of the first temple to our art, the bones of Harris molder—there, like constellations of beacon lights swung out in the realms of mind, are *living* lips—eloquent—almost radiant—with truth and light.

There build the true, solid foundation, and you will sooner or later, bless the day that gave you the resolution.

Finally, let me implore you, if you would reach the Calafornia of true, *enduring* wealth, hesitate not to stem the deep waters, or to struggle over the formidable heights of the isthmus of science.

*Louisville, April* 9, 1866.

